# Digital Approach for the Rehabilitation of the Edentulous Maxilla with Pterygoid and Standard Implants: The Static and Dynamic Computer-Aided Protocols

**DOI:** 10.3390/mps3040084

**Published:** 2020-12-21

**Authors:** Alessio Franchina, Luigi Vito Stefanelli, Simone Gorini, Simone Fedi, Giuseppe Lizio, Gerardo Pellegrino

**Affiliations:** 1 Private Practice, Periodontal and Dental Implant Surgery, 36100 Vicenza, Italy; alessiofranchina@icloud.com; 2 Private Practice, Periodontal, and Dental Implant Surgery, 00145 Roma, Italy; gigistef@libero.it; 3 Private Practice, Prosthodontics, 36100 Vicenza, Italy; gorini.simo@gmail.com; 4 Private Practice, Dental Technician, 51100 Pistoia, Italy; fedisimone@icloud.com; 5 Private Practice, 40125 Bologna, Italy; giuseppelizio@libero.it; 6 Researcher, Oral of Maxillofacial Surgery Unit, Department of Biomedical and Neuromotor Sciences, University of Bologna, Via Massarenti 9, 40138 Bologna, Italy

**Keywords:** fully computer-aided implantology, edentulous maxilla, immediate loading

## Abstract

A full-arch rehabilitation of the edentulous upper jaw without grafting procedures exploits the residual alveolar or the basal bone, with the necessity of long implants placed with a particular orientation. The precision in planning and placing the fixtures is fundamental to avoid clinical problems and to allow an acceptable connection with the prosthesis. The computer-aided implantology resulted in more accuracy than the traditional one, with a high standard of correspondence between the virtual project and the real outcome. This paper reports about the two different digital protocols, static and dynamic, as support to implant-borne prosthetic rehabilitation of edentulous maxillae. Two pterygoid and two/four anterior standard implants were seated in both cases by two different operators, without flap raising, and immediately loaded. This approach avoided the posterior cantilever by-passing the maxillary sinus and was adequately planned and realized without any surgical or prosthetic error. The two digital flow-charts were described step by step, underlining each other’s advantages and drawbacks compared to a free-hand approach.

## 1. Introduction

The digitalization in implant-supported prosthetic rehabilitation is becoming more and more diffuse not only for the planning but even for the operative phase, for transferring the project into the clinical reality [1,2,3,4]. In a fully computerized protocol the Digital Imaging and Communication in Medicine (DICOM) data from a cone beam computerized tomography (CBCT), related to the features of the hard tissue, and the 3D standard tessellation (STL) data, from an intraoral scanner (IOS) detection, related to the teeth and soft tissues surfaces, are paired by a dedicated software; this enables the clinician to choose the implant characteristics and positions according to the anatomical and prosthetic demands [5,6]. After that, to reproduce the virtual plan, the static computer-aided implantology (SCAI) systems or the dynamic computer-aided implantology (DCAI) ones can precisely guide the operator’s hand.

The static system works with a real surgical guide, digitally designed according to the project and printed to be applied in the patient mouth [7,8,9,10,11,12,13,14]. The dynamic computer-aided implantology, on the other hand, works like a Global Positioning System (GPS), in which one or two cameras detect in real-time the spatial relations of reference tools placed on the patient and the surgical handpiece, while software matches this information with CT images and with the superimposed project. This way the clinician can track the drill’s position on 3D radiological anatomy showed on the computer screen while working on the patient [8,9,15,16,17,18]. This protocol implicates high reliability of the involved devices in terms of accuracy [19,20]. In terms of implant positioning precision in comparison with the pre-operative project, the static system and the dynamic one obtained similar outcomes [13,15,16], better to free-hand ones [14]. In any case, both systems enable a mini-invasive and shorter surgery, with flap-less approaches and immediate prosthetic loading.

As far as IOS is concerned, the literature reports a very high precision in the detection of the mouth status in the short span prosthesis. Still, some doubts remain as regards edentulous jaws restoration [3,21]. A Randomized controlled trial recorded no statistical difference between conventional and digital impression for full-arch screw-retained maxillary rehabilitations, with significant less time in the IOS impression group [22]. A precise planning and digitally guided surgery are fundamental for techniques that take advantage of the residual alveolar or basal bone, employing longer fixtures to be inserted in risky anatomic locations with a well-specific orientation: in particular pterygoid implants, for the restoration of atrophic maxillary posterior areas, can damage the great palatine artery and the pterygoid plexus. The full-arch rehabilitation of edentulous upper jaws can be performed with the use of two posterior tilted and two frontal implants [23]. These graft-less procedures, faster and less prone to complications, can be applied even in systemic health critical patients [24,25] The use of bilateral pterygoid implant in a full-arch rehabilitation associated with two-four anterior ones has been adopted with good results, and Stefanelli et al. succeeded in obtaining the full-arch maxillary restorations of thirteen patients with immediate loading and flapless surgery [26]. The use of zygoma implants can be indicated in case of extreme alveolar bone loss, since the difficulties in their placement and loading [19].

This paper aims to describe two specific digital workflows for the immediate rehabilitation of two upper jaws with hopeless residual dentition employing pterygoid and standard implants.

## 2. Procedure

Workflow for SCAI approach.

First step: analysis of oral and general health status of the patient, examination of his requests and the evaluation of several options to solve the patient’s need (new or recent panoramic or periapical radiographs are needed), the explanation of the possible options in terms of the clinical procedures, timing, and costs.

Second step: IOS/conventional impression to prepare a digital wax-up and a related scan prosthesis (to be evaluated in consideration of the span width) and some pictures taking to get the intra- and extra-oral aesthetic requirements.

Third step: a check of the wax-up to evaluate aesthetics, phonetics, and occlusion, and a CBCT to be taken to evaluate the feasibility of that surgical option, including surgical planning.

Fourth step: the surgical guide was designed and printed in resin material according to the implant seating plan.

Fifth step: implant placement with the printed guide fixed in the mouth.

Sixth step: multi-unit abutments (M.U.A.) placement and IOS/conventional impression to manufacture a fixed provisional prosthesis and a silicon bite-check index to find out the correct occlusal height.

Seventh step: provisional prosthesis delivery.

Workflow for DCAI approach.

First, second and third step: the same of the SCAI approach.

Fourth step: insertion of four mini-screws into the alveolar bone functioning as fiducial landmarks for the trace procedure and CBCT taking.

Fifth step: trace (or registration).

Sixth step: implant placement after intra-operatory calibration.

Seventh step: IOS impression and provisional prosthesis delivery.

## 3. Cases Report

### 3.1. SCAI Approach Clinical Case

**Figure 1:**
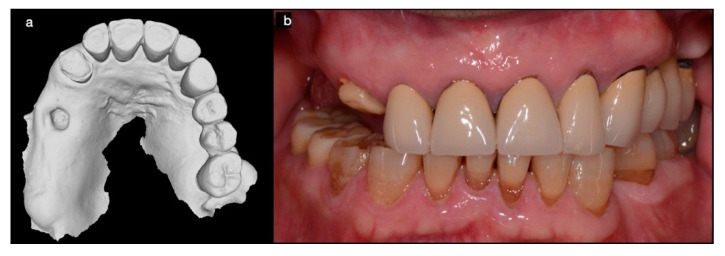
Intraoral digital impression (**a**) and frontal view of the occlusion (**b**).

A 70 years old man, in good general health was referred for the restoration of his maxilla with a fixed full-arch implant-prosthetic rehabilitation. The residual natural dentition resulted in inadequate for valid rehabilitation. Digital workflow has been performed to plan the treatment. A CBCT of both jaws, preoperatory pictures, intraoral scans (Figure 1), and conventional impressions was taken.

**Figure 2:**
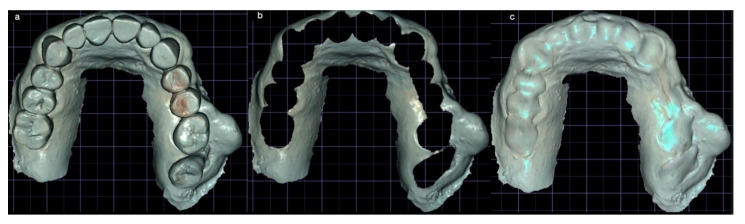
The virtual model obtained after the scanning of the waxed-up plaster cast (**a**). The virtual model after the digital cancellation of the teeth (**b**). The virtual model after the digital filling of the empty spaces (**c**).

A prosthetic wax-up of the maxillary teeth was conventionally performed to verify the correct vertical dimension of the future prosthesis and then was scanned (Figure 2) and the relative STL data were loaded in SCAI software (Exoplan-Exocad, GmbH, Darmstadt, Germany) as well for the DICOM data from the CBCT. Then it was printed with transparent resin to allow clinician making a check after extractions were performed.

**Figure 3:**
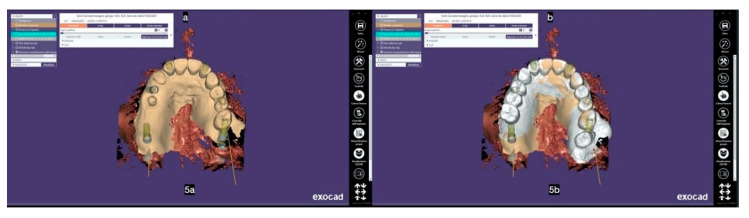
The superimposition of STL files from both intraoral (**a**) and waxed-up plaster model scanning (**b**) onto the DICOM files from the CBCT.

A preliminary modification of the dentate virtual cast was necessary to create a completely edentulous final virtual cast: all the teeth were virtually extracted filling the residual space (Figure 2). After the superimposition of the hard tissue imaging with those related to the teeth and soft tissues before and after the wax-up (Figure 3), two pterygoid implants were planned to be inserted, extending the teeth support until the 2nd molar zone, in addition to two to four implants planned in the area between the two maxillary sinuses.

**Figure 4:**
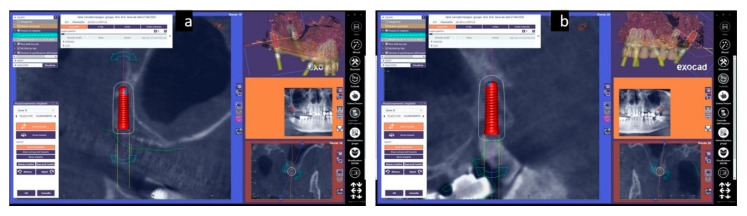
The digital planning of the right (**a**) and left (**b**) pterygoid implants position.

With their angulation, they completely pass the tuberosity before making contact with the lateral wing of the sphenoidal process. This area is rich in cortical bone and its engagement with the implant tip is recommended (Figure 4).

Then the surgical guide was designed and printed in resin material.

**Figure 5:**
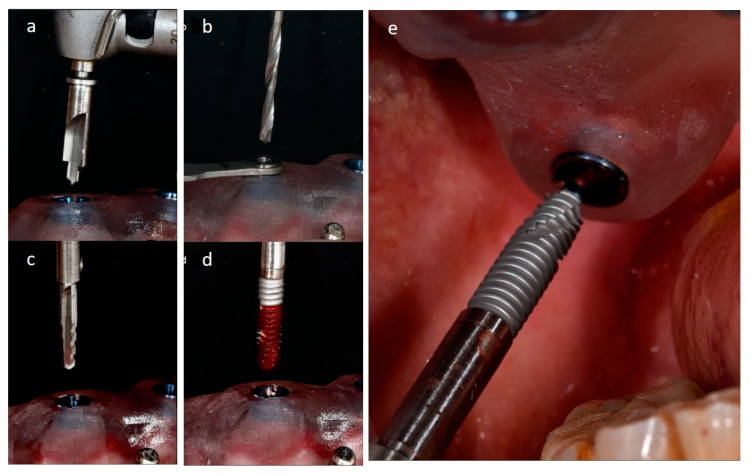
Pterygoid implant beds’ preparation with the printed surgical guide fixed in place: the cortical perforator (**a**), the following twist drill (**b**), the shaping drill burs (**c**), and the implant seating with the implant carrier (**d**,**e**).

During the surgery, all the extractions were carefully carried out to avoid any buccal bone fractures and the guide was fitted and fixed by using four alveolar pins. Each sleeve of the surgical template guided both burs and implant mounters to respectively prepare the bone and to place each implant, accordingly with the planning (Figure 5).

All the fixtures (4.1 mm × 15 mm) were seated and optimal final torque values (>35 Ncm) allowed an immediate loading protocol: at the end of the surgery, low profile abutments were screwed on 1.2 and 2.2 implants (ZimmerBiomet^®^, Palm Beach, Florida, USA) and OT Equators (Rhein 83, Bologna, Italy) on the remaining implants. After one day a full arch temporary prosthesis was delivered.

The prostheses were manufactured before the surgery to reduce the timing of the prosthetic connection.

**Figure 6:**
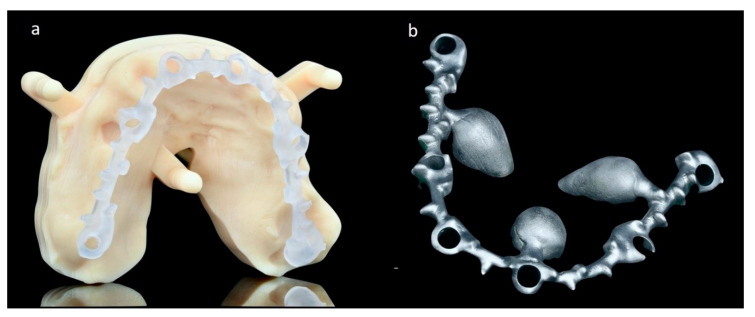
The framework printed in resin after digital planning (**a**) and the metallic melted one (**b**).

The digital workflow allowed a CAD designing of the framework that was printed out with a castable resin and then melted in a metallic bar (Figure 6).

Then, a milled poly-metil-meta-acrylate (PMMA) superstructure was produced to complete the final esthetic and functional coating.

An impression at Low Profile and OT Equator level was taken at the end of the surgical procedure to pour a plaster cast in which the manufactured prostheses with the temporary cylinders was glued.

**Figure 7:**
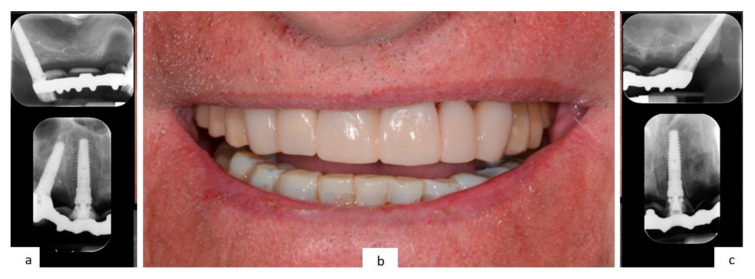
The temporary prosthesis delivered 24 h after surgery (**b**), with the X-rays check of the prosthetic framework fitting (**a**,**c**).

The temporary rehabilitation was delivered and connected within 24 h left from the surgery and a post-operative CBCT was taken to check the final placement of the pterygoid implants. At the same time, periapical X-rays were taken to check the correct fitting of the restoration on each abutment (Figure 7).

### 3.2. DCAI Approach Clinical Case

A 65 years old partially edentulous man with severe mobility of the remaining teeth asked for a full implant-supported prosthetic rehabilitation. A preliminary IOS (Medit i500, Seoul, South Korea) impression was taken to set up a digital wax-up and a CBCT was carried out to evaluate the available bone volume. Before taking the CBCT, four mini-screws were inserted into the alveolar bone functioning as fiducial landmarks for the trace procedure. The so-called “TaP” (Trace and Place) approach was used to perform a dynamic navigation system supported surgery [27]. This consisted of three different steps: Plan, Trace, and Place.

**Figure 8:**
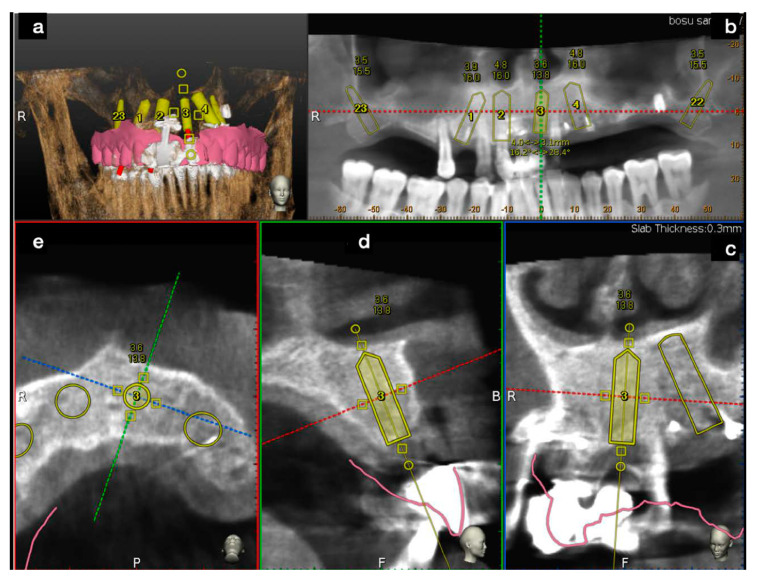
After the wax-up STL’s file was loaded into the surgical software, implants were planned. The software allowed by its windows, a deep check of each implant’s placement, both in the 3D (**a**) view and in the 2D views (**b**–**e**).

First step: Plan. The DICOM data of the CBCT and the digital wax-up were uploaded into the planning software and a prosthetic guided plan for implant characteristics, position, and orientation were accomplished. (Figure 8).

**Figure 9:**
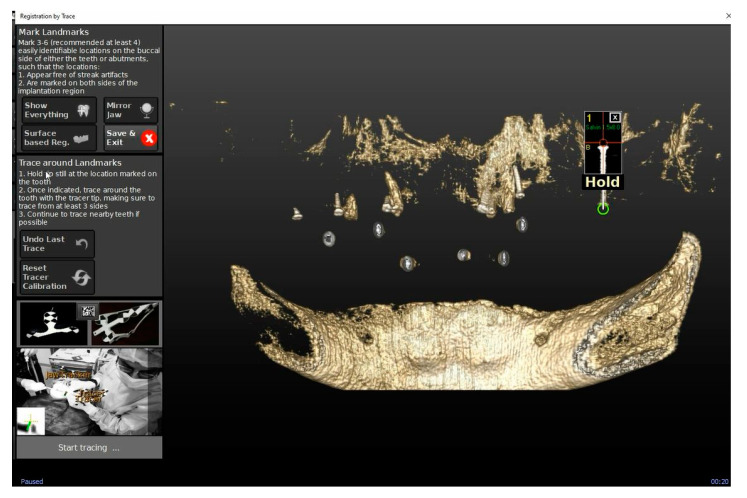
The automatic recognition of the mini screws by the software for the tracing phase.

Second step: Trace (or registration). The system (ClaroNav Inc., Toronto, ON, Canada) associated any point on the patient to the correspondent imaging datum. This phase required a Head Tracker since no residual dentition was available to anchor the Jaw Tracker. The tracing process started at the locations of the fiducial landmarks (the four screws), with the sliding of the tracer ball tip over the surface of each screw. The software automatically recognized the mini-screws and recorded their position after the tracer touched them (Figure 9).

**Figure 10:**
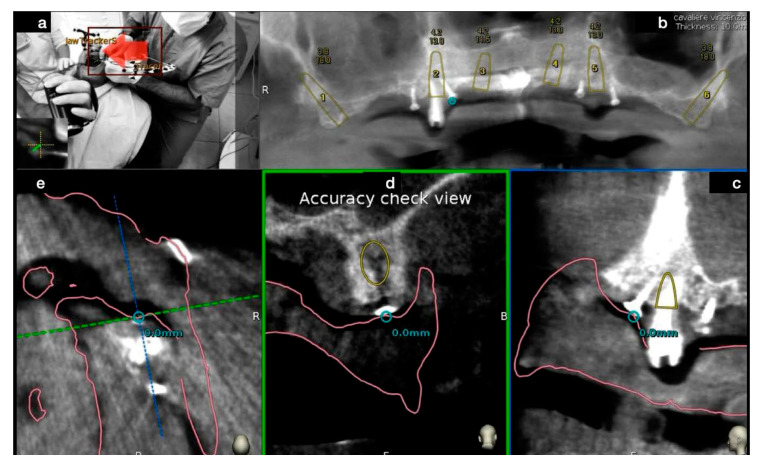
Before starting implant bed’s preparation the operator (**a**) performed an accuracy check of the tracing procedure to verify the congruency between the touched point and its radiological representation, for each available plane (**b**–**e**).

The complete trace and registration process took an average of 1–2 min. The accuracy of this phase is then assessed by touching with tracer’s ball tip any patient’s anatomical marker and confirming congruency between the touched marker and what was shown on the laptop screen (Figure 10).

**Figure 11:**
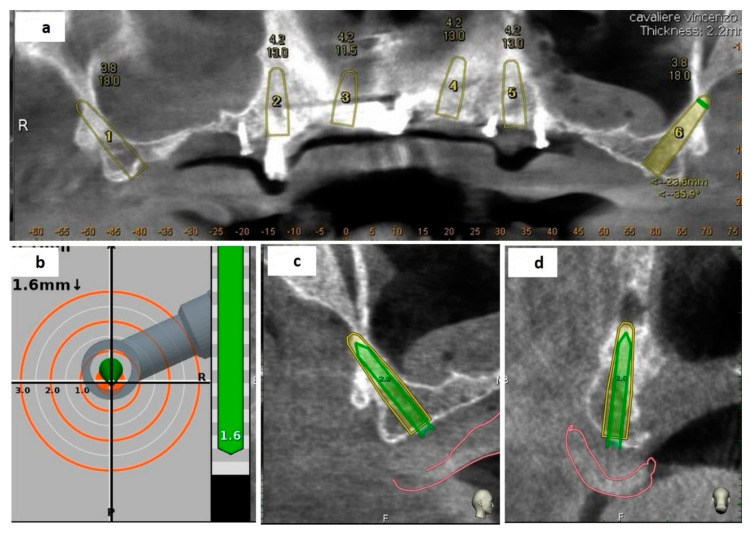
The screen-views during osteotomy: panoramic view (**a**), target view and depth indicator (**b**), mesiodistal section view (**c**), buccal-lingual section view (**d**).

Third step: Place. The axis and the length of the drill and the drill tip length were then calibrated using a metallic caliber; after a second accuracy check, performed in the same manner as for the tracing, the implant placement was performed (Figure 11).

Six implants (A-Z implant, San Lazzaro di Savena, BO, Italy), two pterygoids, and four frontal, according to the so-called “Da Vinci Bridge” protocol, were inserted with a flapless approach. After implant insertion (all implants resulted in torque higher than 35 Ncm, indicating good primary stability), multi-unit abutments (M.U.A.) were selected and screwed onto implants.

**Figure 12:**
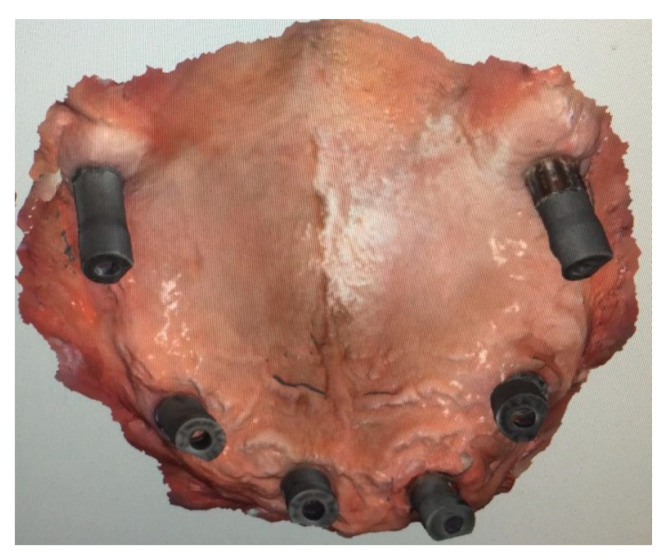
Final impression taken with Intra Oral Scanner (Medit i500, Seoul, South Korea).

A scan abutment (a digital transfer of the abutment 3D position, taken via IOS device) was screwed on each M.U.A., and an the IOS impression (Medit i500, Seoul, South Korea) (Figure 12) was taken to prepare a provisional screw-retained prosthesis.

**Figure 13:**
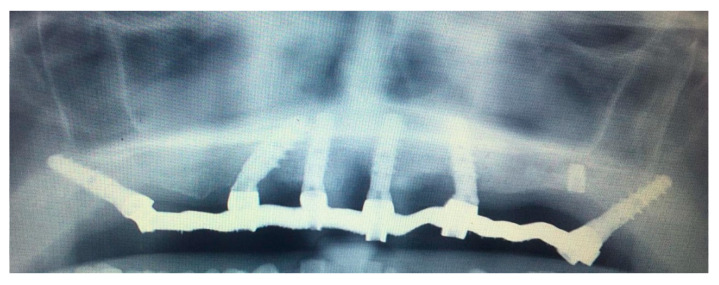
Digital panoramic X-ray showing the correct insertion of the six planned implants and the fit of the temporary prosthesis.

**Figure 14:**
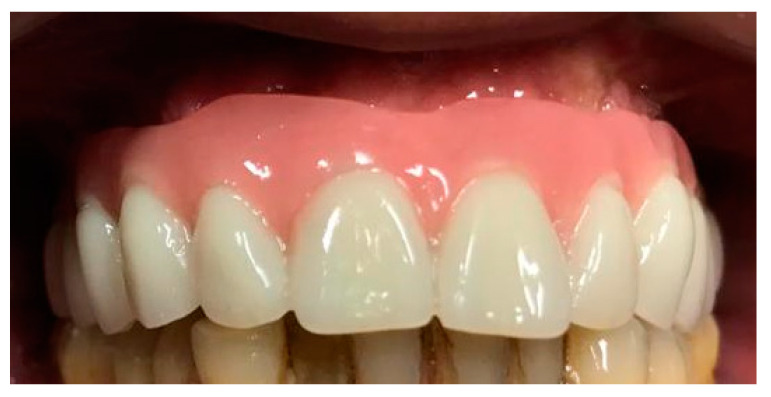
The provisional prosthesis screwed on the M.U.A.

A digitally printed resin template recorded the occlusal relationship between the arches. After 24 h the temporary prosthesis was delivered, an occlusal check was carried out and a final digital panoramic X-ray (Figure 13) was performed to check prosthesis fitting (Figure 14).

In both cases, a nonsteroidal painkiller was prescribed as needed. The patients were instructed to adhere to a soft diet for a few days, to maintain appropriate oral hygiene with daily rinsing using 0.2% chlorhexidine mouthwash, and to gently cleanse with a soft toothbrush, avoiding the use of the floss in the surgical area for the first month postoperatively. No particular post-operative discomfort, such as bleeding, edema, or pain, was complained about by the patients and no complications were recorded.

## 4. Discussion

Full dental rehabilitation of edentulous jaws is challenging for the lack of alveolar bone and teeth [28]. Two possible treatments are considered: the reconstruction of the lost hard tissue before prosthetically driven implant’s placement or the direct implant’s insertion taking advantage of the basal bone [19]. The second approach avoids complex and long-lasting grafting procedures, but it implies the use of longer fixtures to be placed with a particular orientation to reach sufficient primary stability. Such a treatment is strictly dependent on the pre-operative plan’s precision and the reliability of its reproduction in the clinical reality. After all, short implants in the immediate loading full-arch rehabilitation of the maxilla are not investigated for more than five years so far in a few cases [29], even if they have up to an overall of 15-years follow-up [30].

The pterygoid implant, 13 to 20 mm long, works as the distal support for a prosthetic structure in the rehabilitation of posterior maxilla, avoiding any kind of distal cantilever; it by-passes the maxillary sinus and finds the primary stability involving the cortex of the pterygoid process of the sphenoid, the pyramidal process of the palatine bone after passing through the tuber maxillae. Tulasne introduced their use in 1989 to obviate the low density of the bone distally to the sinus [31]. This technique does not depend on the level of atrophy. Rodríguez et al. [32] recorded data from 202 patients and confirmed that an implant of at least 15-mm length should be used to take advantage of the bone’s quantity and quality in this region. Anyway, pterygoid implants did not find a broad consent for the closeness to the greater palatine artery and the pterygoid plexus: a safety distance of at least 3 mm should be kept from the implant’s tip to the greater palatine canal. Furthermore, their inclination implicated an unfavorable emergence profile, with difficulties for the prosthetic connection. Araujo et al. [33] reported from 90% to 98% of survival rate, less than 90% of this datum has been recorded before the functional loading for a lack of osseointegration [34]. Recently, during the 1-year follow-up, high prosthesis stability and no implant loss were observed in 15 patients treated with pterygoid implants and anterior standard ones for edentulous maxillae rehabilitation [35].

The digital support to implantology, strongly encouraged the “pterygoid approach”, particularly with dynamic navigation protocol, with a higher standard of accuracy in implant insertion for partially and for totally edentulous maxillae. Specifically, Stefanelli et al. [34] reported a 50% improved accuracy of the dynamic assisted implantology than free-hand one regarding entry/apical points and apical depth. He reported six times more precision of the digitally supported approach than the free-hand one in terms of degrees of inclination. Concerning the distance kept from the greater palatal canal, the navigated procedure was two times safer than the free-hand one. Besides, authors reported a pre-prosthetic survival rate of 100% versus 93% in the navigated implantology versus a free-hand one, with a substantial reduction of the operative times [23,34]. Regarding statically computed implantology, Vrielinck et al., obtained more accurate results than free-hand approaches only for the parameter of inclination, but worse for those of coronal and apical entry points and, in any case, worse than those with dynamic navigation support [36]. The limited inter-arch dimension in the posterior mouth makes bulky the use of a template that, furthermore, seems to reduce the tactile sensation of the operator, quite useful when the drill and the implant are approaching the pterygoid lamina. Better accuracy in terms of implant placement between SCAI and DCAI was reported in literature only for zygomatic implants [19]. Mediavilla-Guzman et al. recorded better outcomes regarding the angulation parameter with SCAI. Still it is an in vitro study, where the limits related to the use of the guide cannot be adequately considered [37].

Pterygoid implants in a full-arch rehabilitation obtain an optimal distribution of functional loads thanks to a wider implant-prosthetic polygon. Apart from a case report without immediate loading [31], the use of bilateral pterygoid implants associated with two to four standard fixtures in the anterior zone, with a flapless approach, has been reported by Stefanelli et al. [26] for the immediate loading full-arch rehabilitation of the upper jaw. These authors adopted the dynamic navigation implantology after a digital accurate planning to reduce the risk of anatomical injuries and to facilitate prosthetic management. This study reported the accuracy of pterygoid implant insertion of 0.72 mm at the coronal point, 1.25 mm at the apical point, 0.66 mm at apical depth, and 2.86° as an angular deviation. This study’s quite interesting datum was a statistically significant mean difference in accuracy between the frontal implants and pterygoid implants at the apical point and insertion depth: this further confirms the importance of a tutor guide when using longer implants with a particular inclination.

No reports on the use of the static computerized system are readable in literature for a similar technique. Two different operators, each one familiar with the static or dynamic computed system, carried out the herein reported interventions with successful results in both cases. All the advantages of the digital support to implantology, highlighted in the literature, were confirmed with these two cases. Indeed, this report’s clinicians, really expert, could not have otherwise reached all their goals, a fast and harmless flapless surgical procedure with an immediate aesthetic and functional prosthetic finalization, at least with such accuracy. The ND system adopted to treat the herein reported case presents the advantage of accomplishing the registration process by tracing the existing teeth, used as fiducial markers [26]: the presence of stable residual teeth, to be removed because useless for the prosthetic rehabilitation, allows the software to overlap the planning information on the preliminary taken by the patient CBCT, avoiding the need of a new one, that generally must be performed after the insertion of the fiducial markers. This property could not have been exploited in this case report, for the stability reduction of the residual teeth. A real comparison between the two protocols is quite difficult. Both systems can reduce the importance of the operator experience in implantology, even if they requires a familiarity with digital technology and complex prosthetic projects. Regarding the limits of these approaches, apart from the economic costs, the DCAI seems to require a longer ergonomic training to combine the screen’s vision with the surgical field and experienced staff in case of technical problems. On the other hand, a surgical template in SCAI limits the operator’s perceptive feedback, without any possibility of correction of the established plan and to change the implant dimension in the operative phase. The IOS’s use speeds up the treatment timing, eliminating intermediate manual phases and patient discomfort for a conventional impression [38]. This is particularly true using a scanner not needing the powder for the occlusal surfaces’ record as employed herein. Furthermore, the absence of an impression and/or plaster cast lowers the risk of cross-infections and procedural errors. Even in this phase, the practitioner experience seems to play a decisive role in the impression-making process’s precision and duration. Regarding accuracy, it has not been still demonstrated the superiority of the digital approach compared to the conventional one [38]; indeed, Mangano et al. found quite heterogeneous outcomes using twelve different IOS machines taking an impression of six implants inserted in totally edentulous upper arches [21]. Hence, some doubts persist on the IOS’s reliability, particularly when long span prosthesis is going to be realized. Higher accuracy of the digital mock-up, based on a digital optic impression, than the conventional one was reported in literature [39].

Another point to be underlined is the importance of the adopted abutments, M.U.A and OT Equators, in correcting the emergence profile of the pterygoid implants.

Anyway, the new technological frontiers of augmented reality [40] and robotics [41] will improve the digitalization in the dental practice and, in particular, in this type of rehabilitation, whose results have to be confirmed with more cases and longer follow-ups.

## 5. Conclusions

The digital technology, both static and dynamic, hereby reported, helped clinicians to faster the full-arch implant borne rehabilitation of two maxillae minimizing patient discomfort as reported in the literature yet. In particular, the use of CAI helps to transfer the plan to the surgical field without any intra-operatory variations.
